# Structural Determinants of Unique Properties of Human IgG4-Fc^☆^

**DOI:** 10.1016/j.jmb.2013.10.039

**Published:** 2014-02-06

**Authors:** Anna M. Davies, Theo Rispens, Pleuni Ooijevaar-de Heer, Hannah J. Gould, Roy Jefferis, Rob C. Aalberse, Brian J. Sutton

**Affiliations:** 1 Randall Division of Cell and Molecular Biophysics, King's College London, London SE1 1UL, United Kingdom; 2 Medical Research Council and Asthma UK Centre in Allergic Mechanisms of Asthma, London SE1 9RT, United Kingdom; 3 Sanquin Research, Amsterdam 1066 CX, The Netherlands; 4 Academic Medical Centre Landsteiner Laboratory, University of Amsterdam, Amsterdam 1066 CX, The Netherlands; 5 College of Medical and Dental Sciences, School of Immunity and Infection, University of Birmingham, Edgbaston, Birmingham B15 2TT, United Kingdom

**Keywords:** FAE, Fab-arm exchange, IgG4-RD, IgG4-related disease, rFc, recombinant IgG4-Fc, sdFc, serum-derived IgG4-Fc, MES, 4-morpholineethanesulfonic acid, antibody, immunoglobulin, Fab-arm exchange, Fc receptor, C1q

## Abstract

Human IgG4, normally the least abundant of the four subclasses of IgG in serum, displays a number of unique biological properties. It can undergo heavy-chain exchange, also known as Fab-arm exchange, leading to the formation of monovalent but bispecific antibodies, and it interacts poorly with FcγRII and FcγRIII, and complement. These properties render IgG4 relatively “non-inflammatory” and have made it a suitable format for therapeutic monoclonal antibody production. However, IgG4 is also known to undergo Fc-mediated aggregation and has been implicated in auto-immune disease pathology. We report here the high-resolution crystal structures, at 1.9 and 2.35 Å, respectively, of human recombinant and serum-derived IgG4-Fc. These structures reveal conformational variability at the C_H_3–C_H_3 interface that may promote Fab-arm exchange, and a unique conformation for the FG loop in the C_H_2 domain that would explain the poor FcγRII, FcγRIII and C1q binding properties of IgG4 compared with IgG1 and -3. In contrast to other IgG subclasses, this unique conformation folds the FG loop away from the C_H_2 domain, precluding any interaction with the lower hinge region, which may further facilitate Fab-arm exchange by destabilisation of the hinge. The crystals of IgG4-Fc also display Fc–Fc packing contacts with very extensive interaction surfaces, involving both a consensus binding site in IgG-Fc at the C_H_2–C_H_3 interface and known hydrophobic aggregation motifs. These Fc–Fc interactions are compatible with intact IgG4 molecules and may provide a model for the formation of aggregates of IgG4 that can cause disease pathology in the absence of antigen.

## Highlights

• The first high-resolution crystal structures of IgG4-Fc have been solved.
• Arg409 adopts two conformations, each with a different effect on the C_H_3–C_H_3 interface.
• Crystal packing analysis reveals a novel Fc–Fc interface.
• The C_H_2 domain FG loop adopts a unique conformation, affecting FcγR and C1q binding.
• The IgG4-Fc crystal structures explain unique biological properties of IgG4.

## Graphical abstract

**Figure:**
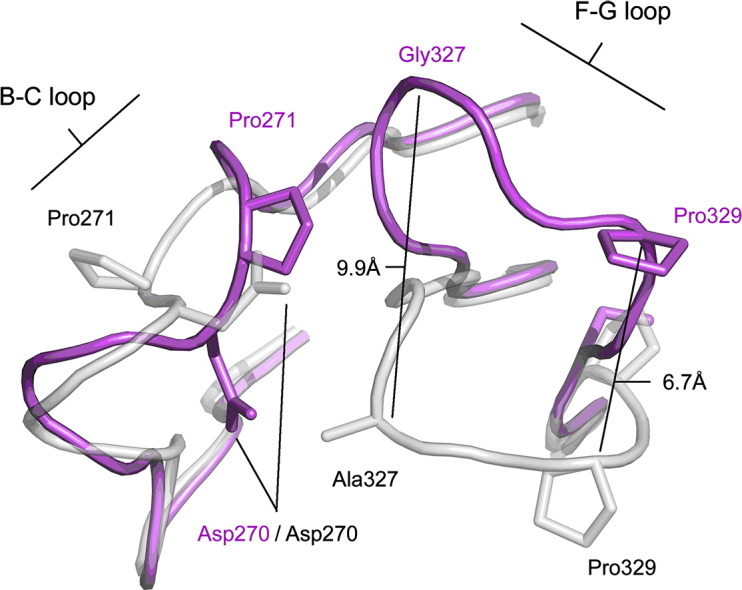

## Introduction

The four subclasses of human IgG display a high degree of sequence homology in their constant regions, yet they play distinct roles and exhibit different patterns of receptor interactions. These functional differences may be attributed in part not only to the lengths and sequences of their hinge regions, but also to subtle sequence variations in the C_H_2 domain [1]. IgG1 and IgG3 play important roles in antibody-dependent cell-mediated cytotoxicity (ADCC) and the activation of complement, through binding to high affinity Fcγ receptors and C1q, respectively [1], [2]. With the exception of FcγRI, IgG4 binds Fcγ receptors with lower affinity [2], and in lacking the effector functions of IgG1 and IgG3, including C1q binding, is considered to be anti-inflammatory [3], [4]; however, a clinical trial involving an IgG4 subclass antibody still gave rise to an unexpected systemic inflammatory response [5].

While IgG4 is normally the least represented IgG subclass in serum, levels are elevated in rheumatoid arthritis [6], IgG4-related disease (IgG4-RD) and auto-immune pancreatitis [7], [8], as well as under conditions of chronic exposure to an antigen, or after allergen-specific immunotherapy [9], [10]. It does however exhibit a unique property, namely the ability for the two heavy (H) chains to disengage, forming a “half-molecule”, and then re-assemble with H chains of another IgG4 antibody, perhaps with different specificity, to form a bispecific antibody that is monovalent with respect to each specificity [9], [11], [12]. This phenomenon, known as Fab-arm exchange (FAE), occurs *in vivo* and is proposed to further contribute to the anti-inflammatory properties of IgG4 [3], [13]. The core sequence of the IgG4 hinge (residues 226–230), which promotes formation of intra- rather than inter-H chain disulfide bonds, and residue Arg409 at the C_H_3–C_H_3 interface, which weakens the non-covalent association between these domains, are requirements for FAE to occur, and dissociation of the C_H_3 domains is a rate-limiting step in the exchange mechanism [3], [14], [15], [16], [17].

Of the four IgG subclasses, IgG1 has been studied most extensively in structural terms. There are crystal structures for human IgG1-Fc alone (e.g., Refs. [18], [19], [20], [21]) and in complex with FcγRII [22], FcγRIII [23], [24], [25], [26], staphylococcal protein A [27], streptococcal protein G [28], TRIM21 (*tri*partite *m*otif *21*) [29], HSV-1 (*h*erpes *s*implex *v*irus *1*) Fc receptor [30] and rheumatoid factor [31], as well as a structure of the whole antibody [32]. More recently, crystal structures for human IgG2-Fc have also been reported [33], [34]. However, despite its use as a therapeutic monoclonal antibody and intriguing biological properties, high-resolution structural information for IgG4 is limited. The structure of IgG4 has been studied in solution [35], [36], and only one low-resolution crystal structure (3.15 Å) of IgG4-Fc in complex with a rheumatoid factor, and one high-resolution crystal structure (1.8 Å) of the isolated C_H_3 domain dimer, have been solved [17], [37].

We report here the crystal structures of IgG4-Fc obtained from papain digestion of IgG4 from patient sera (sdFc), and recombinant IgG4-Fc (rFc), both at substantially higher resolution (2.35 Å and 1.9 Å, respectively) than the previous Fc study. Consequently, we reveal the effects of variation in Arg409 conformations on the C_H_3 interface, providing a better understanding of this controlling factor for FAE. A novel Fc–Fc interaction is also observed that may provide a model for the aggregation of IgG4 in disease, and a general model for IgG aggregation in therapeutic monoclonal antibody preparations. Finally, and unexpectedly, the FG loop in the C_H_2 domain of IgG4-Fc, which in other IgG subclasses is involved in both Fcγ receptor [23], [24] and C1q binding [19], is found to adopt a conformation that would disrupt these activities. Interaction between this loop and the lower hinge region, as in IgG1, would also be impossible, with further implications for FAE. The structural determinants for many of the unusual functional properties of IgG4 are thus revealed in this study.

## Results

### Overall structure and glycosylation

The overall IgG4-Fc domain topology resembles that of other IgG-Fc fragments [1] and residues 236–445 (chain A) and 236–444 (chain B), and 237–444 (chain A) and 238–443 (chain B) were built for the recombinant IgG4-Fc (rFc) and serum-derived IgG4-Fc (sdFc) structures, respectively. The *B*-factors for the C_H_2 domain of chain B in both structures were higher than those for the other C_H_2 and C_H_3 domains, as were those for the N-linked oligosaccharides attached to these two domains. A complex biantennary core was modelled in both structures, and with the exception of chain B of the sdFc structure, a fucose residue, attached to the first *N*-acetylglucosamine residue covalently bound to Asn297, was also built. In both rFc and sdFc structures, the α(1–3) oligosaccharide branch mannose residues form a hydrogen bond with one another.

The sdFc structure was solved using the previously characterised Rea IgG4 myeloma protein [38]. The Rea IgG4 oligosaccharide is a 70% G(0) glycoform that was later enzymatically galactosylated to yield a 100% G(2) glycoform [39]. In both chains, a galactose residue attached to the α(1–6) branch forms hydrogen bonds with surrounding protein residues, but the α(1–3) branch galactose residue was disordered.

The terminal α(1–6) galactose residue is absent in the rFc structure, and instead, additional water molecules fill the space. Terminal galactose is observed in recombinant IgG structures where protein has been produced in CHO cells (e.g., Refs. [26], [30]), and we attribute the absence of galactose in the rFc structure to the use of HEK cells [40]. Apart from this difference in glycosylation, the overall structures of rFc and sdFc are essentially identical.

### Arg409 and the C_H_3–C_H_3 interface

**Fig. 1:**
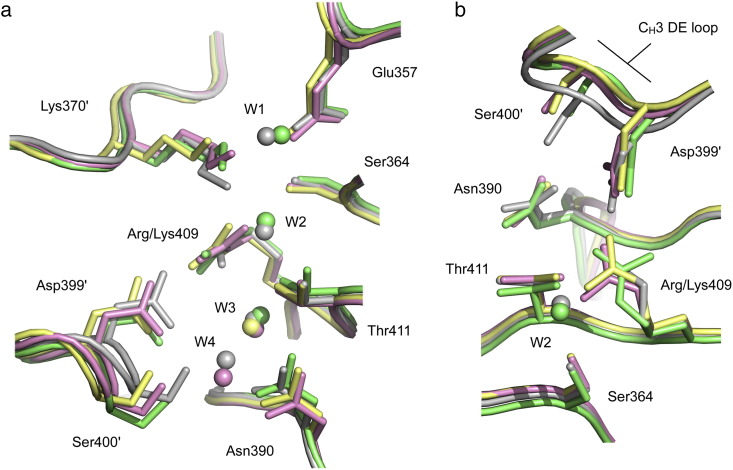
The environment of Arg409 at the C_H_3–C_H_3 interface. (a) Arg409 and nearby residues are shown for the C_H_3 domain dimer structure in pink (PDB ID 4B53 [17]), chain A (green) and chain B (yellow) of the recombinant IgG4-Fc (rFc) structure. IgG1-Fc (PDB ID 3AVE [21]) is coloured grey. Conserved water molecules are labelled W1–W4. Arg409 conformation 2 in the C_H_3 domain and chain B of the rFc structure prevents W2 binding, while conformation 1 in chain A of the rFc structure does not. (b) Alternative view of the two conformations adopted by Arg409 in IgG4. For clarity, Glu357, Lys370′, W1, W3 and W4 are not shown.

Arg409 was recently identified as the key IgG4 C_H_3 domain residue controlling FAE [14]. In IgG1, IgG2 and IgG3, which do not undergo FAE, this residue is lysine. The high-resolution crystal structure of the IgG4 C_H_3 domain dimer showed how Arg409 weakened the C_H_3–C_H_3 interface by disrupting a network of conserved water molecules that mediate inter-domain hydrogen bonds, reducing contact at the edge of the interface through DE loop movement, and lowering its buried surface area [17]. The conformation of Arg409 seen in this high-resolution C_H_3 dimer structure differed from that observed in the earlier IgG4-Fc structure [37], but the low resolution of the latter prevented any conclusions from being drawn concerning conformational variation of Arg409. However, we can now see clearly in the two structures reported here that unlike Lys409 in IgG1, which is conformationally conserved in all known structures, Arg409 can indeed adopt two alternative conformations at the interface, upon which they have different effects (Fig. 1).

In conformation 1, found in the A chains of both rFc and sdFc, as well as the low-resolution Fc structure, the Arg409 guanidinium group is oriented towards Asp399′ (of the other H chain), forming an electrostatic interaction (Fig. 1). In the rFc structure, three conserved water molecules (W1–W3) are present at the C_H_3–C_H_3 interface, while in the sdFc structure, two conserved water molecules (W3–W4) are present, and the hydrogen bond network is comparable to that found in IgG1. The network is also observed in IgG2, which does not undergo FAE, where residue 409 is lysine [33], [34]. Crucially, the rFc structure demonstrates the ability of W2 to bind at the C_H_3–C_H_3 interface in the presence of Arg409. In conformation 2, however, seen in the B chain of rFc and the high-resolution C_H_3 dimer structure, the Arg409 guanidinium group forms hydrogen bonds with both Asp399′ (of the other H chain) and Ser400′. In occupying the location of W2, and affecting the conformation of Lys370′ and the position of W1, conformation 2 disrupts the network of water molecules that mediate inter-domain hydrogen bonds.

On the other hand, in chain B of the sdFc structure, *B*-factors for residues at the C_H_3 domain surface, close to the C_H_3–C_H_3 interface, are considerably higher than those for the rest of the domain. The Ser400′ side chain points away from Asn390, its hydrogen bonding partner, and other interface residues, including Arg409, are partially disordered.

Arg409 conformation 1 is thus more akin to an IgG1-like C_H_3–C_H_3 interface, while conformation 2 clearly weakens the interface through disrupting the water molecule network. The buried surface area for the interface with conformation 2 (calculated for residues in the vicinity of Arg409) is ~ 55 Å^2^ less than that for an interface with Arg409 in conformation 1, which is in turn comparable to that of IgG1.

### Fc–Fc interactions revealed through crystal packing

Interactions mediated through antibody Fc regions, particularly those of the IgG4 subclass, are of interest because they have been implicated in disease-related immune complex formation [6], [7]. Antibody aggregation is also critically important to the therapeutic antibody market, which includes a number of IgG4 monoclonal antibodies [41], [42], [43], [44].

**Fig. 2:**
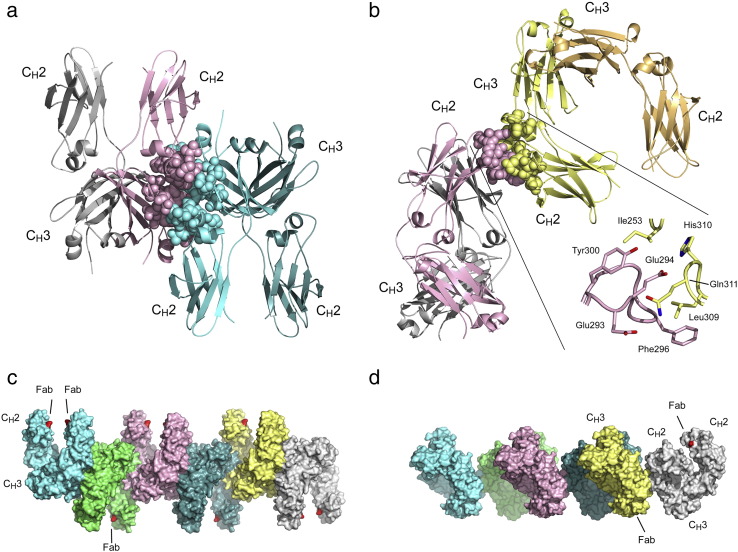
Fc–Fc interfaces identified through crystal packing. (a) Orientation of the two Fc molecules in interface I. The two Fc molecules are related to one another by a twofold rotation. Interfacing residues from one Fc molecule (chain B, grey), and another Fc molecule (chain A, cyan), are depicted as spheres. (b) Orientation of the two Fc molecules in interface II. The top of the C_H_2 domain from one Fc molecule (chain A, pink) interacts with the side of the C_H_2 domain from another molecule (chain A, yellow), close to the C_H_2–C_H_3 interface. Interfacing residues are depicted as spheres. Inset: Detailed view of the interface, showing residues Glu293, Glu294, Phe296 and Tyr300 from the first molecule (pink), and residues Ile253, Leu309, His310 and Gln311 from the second (yellow). For clarity, Gln268 is not shown. (c) Fc molecules interacting through interface I can be assembled in a linear fashion. In each chain, residue 327 at the N-terminus of the C_H_2 domain, where the Fc region is connected in turn to hinge and Fab regions, is coloured red. Fab regions would be oriented alternately above and below the plane of assembly. (d) An assembly of Fc molecules created by interface II. The assembly is formed from interactions between chain A of successive Fc molecules. Once again, residue 327 is coloured red, and the approximate position of Fab regions from two adjacent molecules is indicated.

Crystal contacts in the IgG4-Fc structures were analysed, and two extensive interfaces were identified (Fig. 2a and b). Interface I (Fig. 2a) comprises two Fc molecules related by a twofold rotation, buries a surface area of ~ 1480 Å^2^ and involves contact between C_H_2 and C_H_3 domains of different chains. Van der Waals interactions predominate, and there is a salt bridge (Arg255–Glu382), all of which are duplicated due to the twofold nature of the interaction. A PISA [45] search of the Protein Data Bank (PDB) revealed a similar twofold related interface in other IgG1-Fc structures, with buried surface areas ranging from 1020 Å^2^ to 1990 Å^2^ but virtually identical orientations of the Fc pairs.

Interface II (Fig. 2b) involves the “top” of the C_H_2 domain of one Fc molecule, close to Asn297 to which oligosaccharide is attached, interacting with the “side” of the C_H_2 domain of a second Fc molecule, close to the C_H_2–C_H_3 interface; the overall orientation of the two IgG4-Fc molecules resembles the interaction between IgG-Fc and FcRn [46]. The buried surface area is ~ 860 Å^2^ and hydrophobic interactions predominate [e.g., Phe296 and Tyr300 of one chain (the “top”) and Leu309 and Ile253 (the “side”) of the other; Fig. 2b, inset], although there are also hydrogen bonds (e.g., Glu293–Gln311 and Glu294–His310; Fig. 2b, inset). To our knowledge, interface II has not been observed in any human IgG-Fc structure reported to date, although Fc–Fc interfaces involving residues 253 and 296 have been observed in rabbit and mouse IgG-Fc (Fig. S1) [47], [48].

### The IgG4 C_H_2 domain FG loop adopts a different conformation

**Fig. 3:**
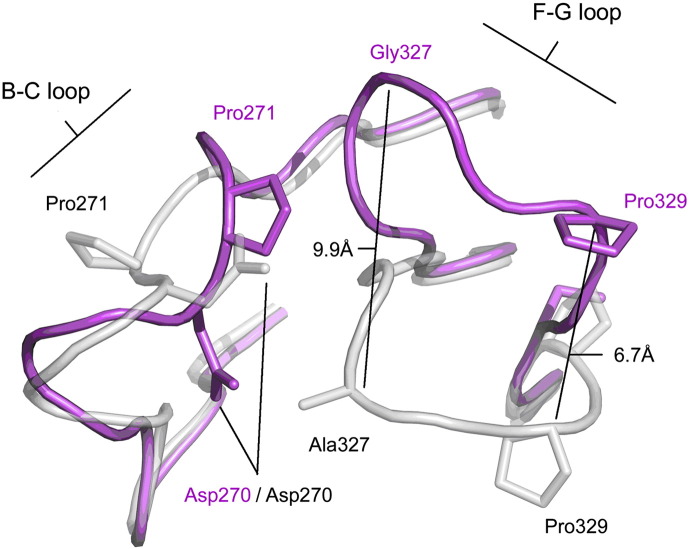
Conformational differences in the C_H_2 FG loop. Superposition of the recombinant IgG4-Fc (rFc) structure (purple) on that of IgG1-Fc (grey) (PDB ID 3AVE [21]) reveals the unique FG loop conformation found in IgG4. The distance between Pro329 (C^α^ atoms) in the two structures is indicated, as well as that for Ala327 (IgG1) and Gly327 (IgG4), highlighting the extent of the differences. The effect on the positions of BC loop residues Asp270 and Pro271 is also clear.

When compared with high-resolution IgG1-Fc structures (e.g., Refs. [19], [20], [21]), the FG loop in both C_H_2 domains of IgG4-Fc is seen to adopt a quite different conformation between residues Ser324 and Ser331 (Fig. 3). This difference folds the FG loop away from the C_H_2 domain to the extent that the C^α^ atoms for residues 327 and 329 differ by 9.9 Å and 6.7 Å, respectively, compared with IgG1-Fc. The conformation is essentially identical to that observed in one domain of an IgG2-Fc mutant structure (the top portion of the other C_H_2 domain was disordered), where the IgG4-Fc C_H_2 FG loop was recreated through two point mutations [34].

However, comparison of main chain φ and ψ torsion angles reveals that values are only substantially different for two residue pairs, 326 and 327 (Lys–Ala in IgG1, Lys–Gly in IgG4) and 330 and 331 (Ala–Pro in IgG1, Ser–Ser in IgG4), while values for Ser324, Asn325, Leu328 and Pro329 (all of which are conserved in IgG) are comparable. That some local structural aspects of the loop are retained implies that these key amino acid differences between IgG1 and IgG4, Pro331Ser and Ala327Gly, both of which introduce conformational flexibility into the loop, are responsible. The adjacent BC loop is, unsurprisingly, affected by the FG loop changes, and residues 270–272 also adopt a different conformation in IgG4 (Fig. 3).

The FG loop conformation in IgG4 is not an artefact of crystal packing. Despite their different packing environments, the conformation is the same in both chains. While an IgG1-like conformation in chain B would clash due to crystal contacts, in chain A, the FG loop is free to adopt either conformation.

### C_H_2 domain FG loop conformation modulates the Fcγ receptor interaction

**Fig. 4:**
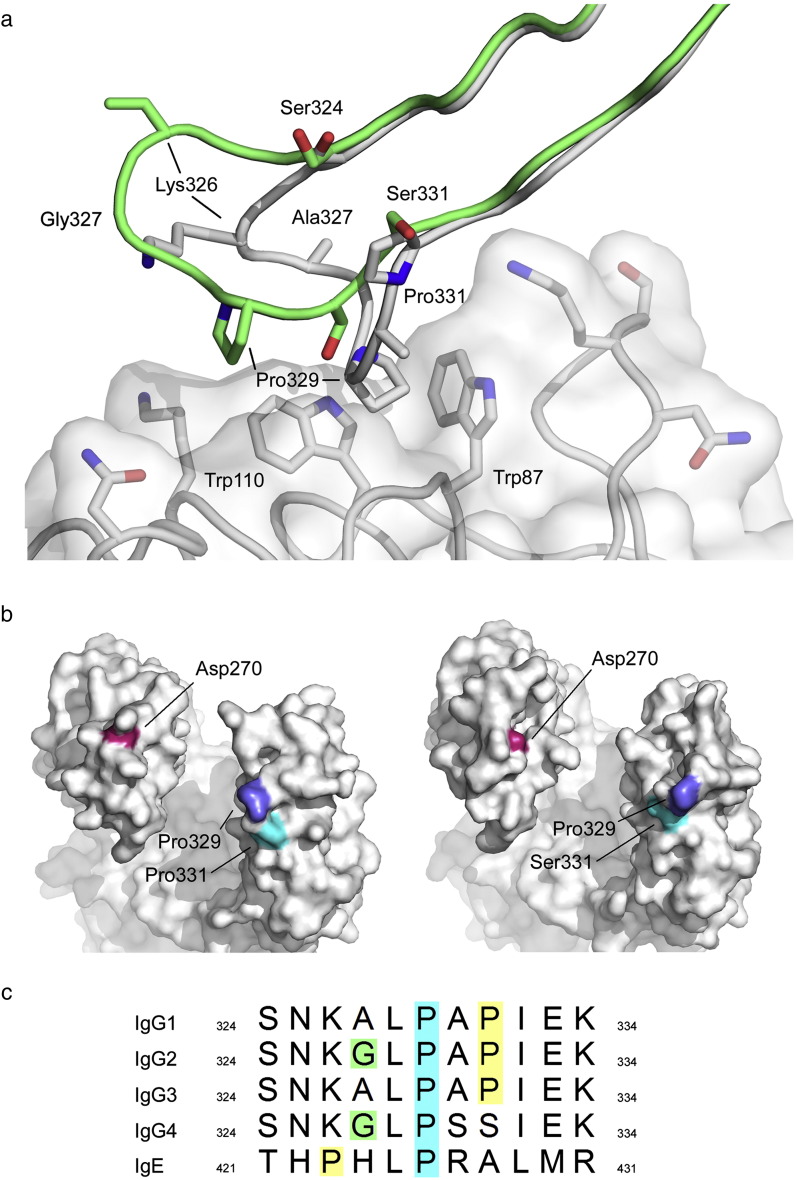
Unique conformation of the C_H_2 FG loop in IgG4-Fc. (a) Superposition of the recombinant IgG4-Fc (rFc) structure (green) on that of IgG1-Fc bound to FcγRIII (grey) (PDB ID 1E4K [23]) reveals how the different conformation of the FG loop in IgG4 cannot interact with receptor through the proline sandwich (Trp87-Pro329-Trp110). For clarity, residues 325 and 328 are not shown. (b) Surface views of IgG1-Fc (PDB ID 3AVE [21]) (left) and IgG4-Fc (right) highlighting residues important for C1q binding to IgG. The positions of residues Asp270 (pink) and Pro329 (blue) are significantly altered, while that of Pro/Ser331 (cyan) is not. Asp270 from chain A, and Pro329 and Ser331 from chain B, are coloured in accordance with a proposed model of the IgG-Fc/C1q interaction [51]. (c) Sequence alignment of human IgG1–IgG4 and IgE for residues comprising the FG loop. The conserved proline (329 in IgG/426 in IgE) involved in receptor binding is highlighted in blue. With the exception of IgG4, all other FG loop sequences contain a second proline residue (yellow) near the proline involved in receptor binding. IgG2 and IgG4 are the only sequences containing glycine at position 327 (green).

The FG loop in the C_H_2 domain is a major determinant of FcγR binding in IgG1 and, together with residues of the lower hinge region, constitutes one of two sub-sites. Pro329 forms a hydrophobic “proline sandwich” with two tryptophan residues in the receptor, a feature seen in both IgG1-FcγRII [22] and IgG1-FcγRIII [23], [24], [25], [26] crystal structures, and the same interaction is conserved (with Pro426) in the IgE-FcεRI complex [49]. The FG loop conformation in IgG4-Fc shifts the position of Pro329 considerably (Fig. 4a). In IgG1, the two sites of receptor engagement, along with the lower hinge region, bury a surface area of ~ 1620 Å^2^ (~ 1170 Å^2^ without the lower hinge region). In moving away from the C_H_2 domain, the different FG loop conformation in IgG4 is unable to contact the receptor, with the loss of ~ 150 Å^2^ buried surface area from the interface.

### C_H_2 domain FG loop conformation alters the C1q binding site

In human IgG1, residues Asp270 (BC loop), Lys322 (strand F), and Pro329 and Pro331 (FG loop) have been implicated in C1q binding [19], [50], [51]. In IgG4, the position of the conserved Lys322 is the same as in IgG1, but Ser331 replaces Pro331 (Fig. 4a), and although conserved, both Asp270 and Pro329 occupy very different positions due to the unique conformation of the FG loop, and the effect that this has upon the adjacent BC loop (Fig. 3, Fig. 4b).

Comparison of the FG loop sequences of human IgG1–IgG4 (Fig. 4c) indicates that a striking, and distinguishing feature of IgG4 in this region, is the Pro to Ser replacement at position 331. Indeed, its role in complement binding has been reciprocally demonstrated experimentally: the mutation Ser331Pro in IgG1 and IgG3 reduces C1q binding [19], [50], while the Pro331Ser mutation partially restores C1q binding to IgG4 [50], [52]. However, an IgG4-like FG loop conformation was not observed in an IgG1-Fc crystal structure containing the Pro331Ser mutation, as crystal packing precluded the formation of such a loop structure [53].

### No contact predicted between the C_H_2 FG loop and the lower hinge/C_H_2 domain junction

The lower hinge is not always fully ordered in IgG1-Fc crystal structures, but Pro238, at the junction with the C_H_2 domain, forms van der Waals interactions with Leu328 from the C_H_2 FG loop. In the few crystal structures of whole IgG reported to date, residues of the FG loop are found to interact with residues close to, or within, the lower hinge (residues 231–237), although the extent of interaction varies due to conformational asymmetry in the crystals. In human IgG1 [32], Leu328 and Pro329 from the FG loop form van der Waals contacts with Gly236 and Pro238 on one chain, and Leu235 and Pro238 on the other. In mouse IgG2 [54], one chain has limited contact between the FG loop and the lower hinge region, but in the other, the two are intimately associated, with Leu328, Pro329 and Ala330 contacting Pro232, Asn233, Leu235 and Pro238. In whole IgG4, the altered FG loop conformation would preclude any contact with either the lower hinge region or Pro238, with potential structural and functional implications.

## Discussion

### Overall structure

The structures reported here of recombinant (rFc) and serum-derived (sdFc) IgG4-Fc represent the first high-resolution crystal structures for this antibody subclass. The overall structure of the Fc fragment is similar to that already reported for the highly homologous IgG1 and IgG2 subclasses, but unique structural features of IgG4-Fc have now been revealed.

### Implications for FAE

The rFc and sdFc IgG4 structures have revealed how Arg409 can adopt two different conformations, each of which affects the C_H_3 interface differently. While conformation 1 is similar to that seen in IgG1 structures, conformation 2 weakens the interface by disrupting the water structure. With the exception of chain B of the sdFc structure where Arg409 is disordered, Arg409 adopts conformation 1 in three chains (PDB ID 1ADQ [37]; rFc chain A and sdFc chain A) and conformation 2 in three chains (PDB ID 4B53; chains A and B [17] and rFc chain B). Only conformation 2 was observed in the C_H_3 domain crystal structure, but overall, the two Arg409 conformers are equally populated.

Intriguingly, the high *B*-factors for residues at the C_H_3 domain surface, and partial disorder at the C_H_3 interface in chain B of the sdFc structure, may represent the transition of Arg409 between the two conformations, or may even provide an early snapshot of the dissociation process itself.

The altered C_H_2 domain FG loop conformation may also play a role in FAE. Isomerisation from inter- to intra-chain disulfide bonds in the hinge region are believed to be a prerequisite for this process, and may be facilitated by flexibility in the core hinge, arising from a Pro (IgG1)/Ser (IgG4) substitution at position 228. The IgG4 C_H_2 FG loop precludes any contact with the lower hinge, and a more mobile lower hinge could in turn enhance flexibility in the core hinge.

Low-angle X-ray solution scattering data for whole IgG4 revealed a change in overall structure from a symmetric shape to an asymmetric shape when Ser331 from the C_H_2 FG loop was mutated to proline [35]. Given the potential for contact between the lower hinge and the FG loop, it is thus possible that overall antibody structure, and function, might be linked to the FG loop conformation.

### Fc–Fc interfaces I and II utilise conserved “consensus binding site” residues

Analysis of interfaces between IgG-Fc and various protein binding partners, FcRn [46], rheumatoid factor [37], protein A [27] and protein G [28], led to the identification of a common “consensus binding site” comprising residues Met252, Ile253, Ser254, Asn434, His435 and Tyr436, from both C_H_2 and C_H_3 domains, located on the side of IgG-Fc [18]. Both interfaces I and II utilise these consensus residues: interface I incorporates residues 254, 434 and 436, while interface II incorporates residue 253. In the IgG4-Fc crystals, both interfaces I and II exist simultaneously; that is, one Fc chain utilises the consensus site to engage two others.

Interfaces I and II share similarities with consensus site residue interactions. In interface II, a hydrogen bond is formed with the backbone of Ile253, as in FcRn, rheumatoid factor, protein A and protein G interactions. Additionally, in interface II, Ile253 is surrounded by Gln268, Glu294 and Tyr300, similar to the interface with FcRn (Glu135, Trp133) and protein A (Gln128, Gln129, Phe132). In interface I, Ser254 forms van der Waals interactions with Tyr436, similar to the interface with protein G (Ser254/Tyr45) and rheumatoid factor (Ser254/Trp52A). Thus, interfaces I and II share generally conserved features of interfaces between IgG and a variety of protein binding partners. (While not forming part of the consensus site, Leu309 is also a contact residue for protein A [27] and rheumatoid factors [55] and, in IgG4, is the site of a leucine/valine isoallotypic variation [56].)

### Residues from Fc–Fc interface II belong to aggregation-prone “motifs”

A recent computational approach identified hydrophobic “motifs” in IgG1 C_H_1, C_H_2 and C_H_3 domains and hinge region, which are prone to aggregation [42]. Motifs involving residues Ile253, Tyr296 (Phe296 in IgG4) and Leu309, all involved in IgG4 Fc–Fc interface II, were among those identified in the study. The same authors found that mutating certain hydrophobic residues, for example, Ile253 and Leu309, to a hydrophilic lysine, indeed improved antibody stability [57].

### Interfaces I and II provide clues about antibody–antibody interactions

With a buried surface area of 1480 Å^2^, Fc–Fc interface I, observed in both IgG1 and IgG4, is comparable with those for Fc binding to the neonatal IgG receptor, rheumatoid factor, protein A, protein G, FcγRIII and HSV-1 receptor (which range from 1350 Å^2^ to 2000 Å^2^). IgG4 Fc–Fc interface II is smaller (860 Å^2^), but could form under certain conditions such as high concentration. IgG Fc–Fc interactions have been documented *in vitro* [58], and *in vivo* in diseases such as rheumatoid arthritis [6] and auto-immune pancreatitis [7], where IgG4 antibody levels are elevated [6], and there is also some evidence that therapeutic monoclonal IgG4 antibodies have a higher tendency to aggregate, compared with IgG1 [44]. However, the equivalent residue to Phe296 from IgG4 is Tyr296 in IgG1, suggesting that a similar interface could also form in this subclass. The concentrations required for therapeutic antibodies, which include both IgG1 and IgG4 [41], may promote such an interaction.

Assemblies using a whole antibody structure [54] as a template can be generated using IgG4-Fc interfaces I and II, in a manner that can also accommodate both Fab regions (Fig. 2c and d, Fig. S2). Novel interfaces, such as those observed in human IgG4, and previously described for mouse IgG2 [48], may therefore indeed be relevant to understanding, and thus preventing, aggregation of whole IgG antibodies.

### The C_H_2 FG loop conformation is unique to IgG4

A DALI structural similarity search [59] of the PDB, using IgG4-Fc as the search term, returned over 150 hits for immunoglobulin heavy chains, including IgG (human, rabbit, mouse, rat), IgE (human), IgA (human), IgY (chicken) and IgM (mouse) isotypes. In the structures of IgG and IgE, isotypes known to engage receptor through a “proline sandwich” interaction, the conformation of the Cγ2 and Cε3 domain FG loop was broadly similar to that found in IgG1, despite crystal packing in the majority of cases that would allow an IgG4-like conformation to be adopted. In only a few heavy chains were slightly different FG loop conformations found, and with the exception of an IgG2 mutant, discussed below, none were similar to that found in IgG4, and the relative position of receptor binding Pro329 (Pro426 in IgE) was unaffected. The structural conservation of the FG loop conformation in IgG1 and IgE thus reflects its role in receptor binding. However, in an IgG2-Fc mutant, in which Ala330 and Pro331 were both mutated to serine [34], the FG loop adopts a conformation similar to that found in IgG4. Since IgG2 naturally has a glycine at position 327 (Fig. 4c), these two additional mutations essentially recreate the IgG4 C_H_2 FG loop in IgG2.

We next examined FG loop conformation in the other immunoglobulin isotypes returned by the DALI search. IgA engages FcαRI at a site located at the Cα2–Cα3 interface, and not through the Cα2 domain FG loop, and the structurally equivalent residue to the receptor binding proline in IgG1 and IgE is lysine [60]. Nevertheless, despite this key difference, and some local perturbation about the lysine residue, the overall conformation is essentially similar to the C_H_2 and Cε3 domain FG loops in IgG1 and IgE, respectively. Structural conservation of the Cα2 FG loop, discussed below, is attributed to two proline residues, which flank the loop on either side.

Less is known about the interactions between IgY and IgM and their receptors. IgY, found in birds and reptiles, engages one receptor, CHIR-AB1, at the Fcυ3–Fcυ4 domain interface [61], [62]. The Fcυ3 domain FG loop was later identified as a binding site for a second receptor, ggFcR [63]. The conformation of the IgY Fcυ3 domain FG loop is essentially identical to that found in IgG1 and IgE [64], and the position of Pro439, equivalent to Pro329 (IgG) and Pro426 (IgE), suggests engagement in a manner similar to that of IgG/FcγR and IgE/FcεRI. The recently published NMR structure of the mouse IgM Fcμ3 domain also revealed an FG loop with a similar conformation to those found in IgG1, IgE and IgY [65]. Thus, to the best of our knowledge, among native antibody isotypes, the IgG4 C_H_2 FG loop adopts a unique conformation.

A common feature of these structurally conserved FG loops is the presence of two proline residues. In IgG, IgE, IgY and IgM, the first, receptor binding (or putative receptor binding), proline residue is found at position 329, 426, 439 and 312, respectively. IgG, IgY and IgM have a second, structurally equivalent proline residue at position 331, 441 and 314, respectively. In IgE, this residue is alanine, and a second proline residue is instead found at position 423. IgA does not have a receptor binding proline residue, and the overall conformation of the Cα2 FG loop is instead maintained by two flanking proline residues. On one side of the loop, Pro333 is structurally equivalent to Pro331 from IgG1, Pro441 from IgY and Pro314 from IgM, while on the other, Pro328 is structurally equivalent to Pro423 from IgE. IgG4 does not have a second proline residue located within the C_H_2 domain FG loop, and the implications of this are discussed later.

### Does the C_H_2 FG loop modulate receptor binding in IgG4?

**Fig. 5:**
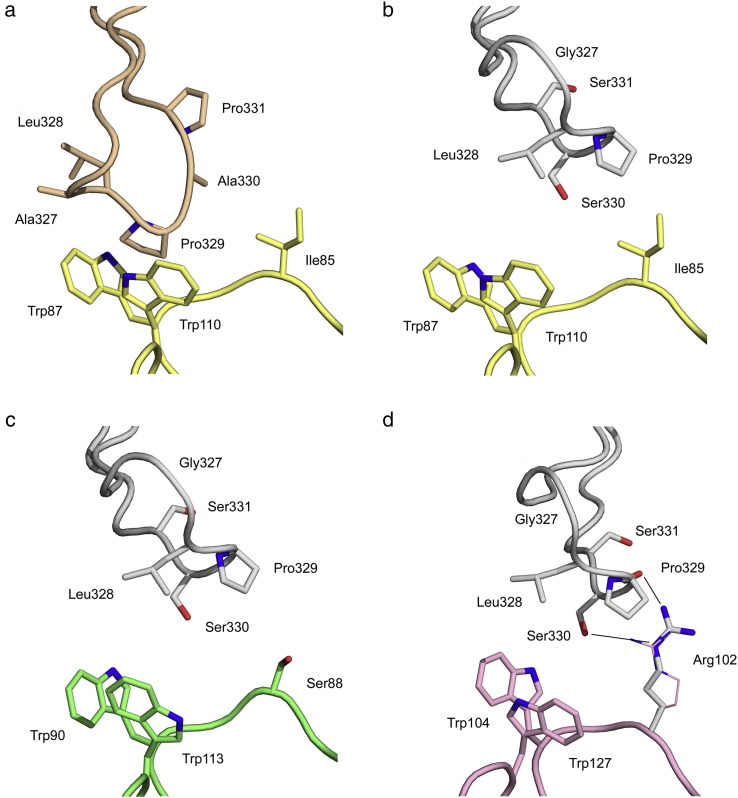
The C_H_2 FG loop and Fcγ receptor binding via the proline sandwich. (a) IgG1 FG loop (orange) forms a proline sandwich with FcγRIII (yellow) (PDB ID 1E4K [23]). (b) IgG4-Fc (grey) superposed onto IgG1-Fc from PDB ID 1E4K demonstrates that the proline sandwich interaction with FcγRIII (yellow) is disrupted, and there would be no interaction between the IgG4 FG loop and FcγRIII.(c) Comparison with the structure of IgG1 in complex with FcγRII (PDB ID 3RY6 [22]) reveals that there would be no interaction between the IgG4 FG loop (grey) and FcγRII (green). (d) Superposition of IgG4-Fc (grey) onto IgG1-Fc from PDB ID 1E4K, and FcγRI (pink) (PDB ID 3RJD [70]) superposed onto FcγRIII from PDB ID 1E4K, reveals that, with a change in conformation, Arg102 could form two hydrogen bonds, indicated with black lines, with Pro329 and Ser330 from the IgG4 FG loop. The hydrogen bond with Pro329 is preserved in IgG1 and IgG4/FcγRI interactions while that with Ser330 is unique to IgG4. The conformation of Arg102 found in the FcγRI structure is shown in pink, and the modelled conformation is in grey.

A strictly conserved feature of the C_H_2 domain FG loop in IgG is a proline residue at position 329, which is important not only for receptor interaction, but also for C1q binding (Fig. 4c). In IgG1, mutation of Pro329 to alanine alone significantly reduces binding to all Fcγ receptors and C1q [19], [66]. In IgG3, mutation of Pro331 to serine reduces affinity for receptor [67]. The disrupted proline sandwich interaction, caused by the different C_H_2 FG loop conformation, is certainly consistent with the lower affinity of IgG4 for FcγRIIa (*K*_A_ of 1.7 × 10^5^ M^− 1^ and 2.1 × 10^5^ M^− 1^ for the H131 and R131 variants, respectively) and FcγRIIIa (*K*_A_ of 2.0 × 10^5^ M^− 1^ and 2.5 × 10^5^ M^− 1^ for the F158 and V158 variants, respectively), compared with IgG1 [*K*_A_ values of 5.2 × 10^6^ M^− 1^ (FcγRIIa, H131), 3.5 × 10^6^ M^− 1^ (FcγRIIa, R131), 1.17 × 10^6^ M^− 1^ (FcγRIIIa, F158) and 2.0 × 10^6^ M^− 1^ (FcγRIIIa, V158)] [2]. Modelling of the IgG4-Fc structure onto the crystal structures of the IgG1-Fc complexes with FcγRII and III illustrates this clearly (Fig. 5a–c).

However, interaction between IgG4 and the lower affinity FcγRII and FcγRIII receptors is further complicated by differences between receptor variants. While IgG4 binds FcγRIIIa, it does not bind FcγRIIIb [2]; residues for these two receptors are identical in the immediate vicinity of the proline sandwich, and the contrasting affinity is attributed to sequence differences at positions 147 and 158 [2]; critically, position 147 is located at the second receptor binding sub-site on the other C_H_2 domain. Furthermore, IgG4 binds FcγRII receptors with lower affinity, but FcγRIIb is bound with higher affinity than FcγRIIa [2]. An arginine residue is found at position 131 in FcγRIIb, whereas in FcγRIIa, the equivalent residue is histidine. Incidentally, an arginine/histidine difference at this position is also responsible for the high- and low-responder forms of FcγRIIa [68]. Of the FcγRII receptor variants, the low-responder form of FcγRIIa, with histidine at position 268, is bound by IgG4 with the lowest affinity. Intriguingly, in the IgG1-Fc/FcγRIIa crystal structure, Arg131 from the receptor lies close to residue 268 from the BC loop [22], which is histidine in IgG1 and glutamine in IgG4. If Arg131 were to adopt a different conformation, the higher affinity of IgG4 for FcγRII variants with arginine at this position could, in part, be explained by a hydrogen bond with Gln268 from the BC loop.

The affinity of IgG4 for FcγRI is not only higher than that for FcγRII and FcγRIII, but of the same order of magnitude as that for IgG1 (*K*_A_ of 6.5 × 10^7^ M^− 1^ and 3.4 × 10^7^ M^− 1^ for IgG1 and IgG4, respectively) [2]. In contrast to FcγRII and FcγRIII, where IgG immune complexes are responsible for receptor interaction, monomeric IgG4 is responsible for the interaction with FcγRI [2], [69]. Although there is no crystal structure for the IgG1-Fc complex with FcγRI, a model based upon the FcγRI crystal structure has been proposed [70], and if IgG4-Fc is substituted for IgG1-Fc in this model, a hydrogen bond, additional to that between Arg102 and Pro329 (main chain), may be made between Arg102 of FcγRI (which is unique to this receptor) and Ser330 (side-chain) in the FG loop conformation of IgG4 (Fig. 5d).

Could the additional hydrogen bond between Arg102 and Ser330 side chains compensate in the absence of a proline sandwich in the IgG4/FcγRI receptor interaction, allowing IgG4 to bind with an affinity comparable to that of IgG1? The FcγRI crystal structure suggested that the presence of a tyrosine residue at position 176 in FcγRI, rather than the structurally equivalent valine in FcγRIII, created an additional hydrogen bond between FcγRI and the lower hinge that could account for the difference in isotype specificity and, therefore, affinity, between FcγRI and FcγRIII for IgG4 [70].

In summary, the altered C_H_2 FG loop conformation in IgG4 cannot solely account for the differences in affinity between IgG4 and Fcγ receptors, but conformational variation in the loop may act together with sequence differences between the Fcγ receptors, in addition to alternative interactions with the lower hinge, to modulate IgG4 affinity. It remains to be determined whether, unaffected by crystal packing restrictions, the native IgG4 loop could also adopt the conserved IgG1-like conformation, potentially adding a further level of complexity in control of receptor affinity.

### Which residues are responsible for the different C_H_2 FG loop conformation in IgG4?

Point mutations have confirmed the role of FG loop residues in controlling IgG function, and two decades ago it was speculated that sequence variation in this region might affect FG loop conformation [71]. IgG4 does not possess a second proline residue in its FG loop (Fig. 4c) and Pro331 is replaced by serine. Furthermore, IgG4 is different from IgG1, IgG3 and IgE in that, like IgG2, it has a glycine residue located within its FG loop, at position 327. The crystal structure of IgG2-Fc revealed no effect on FG loop conformation due to packing constraints [33]. Likewise, the structure of an IgG1-Fc mutant engineered to remove effector functions, containing a Pro331Ser mutation, revealed no effect on the FG loop, but once again, steric restrictions due to crystal packing precluded formation of an IgG4-like FG loop conformation [53].

A recent structural study of an IgG2-Fc mutant [34] led to the suggestion that the altered position of Pro329 ultimately resulted from disruption of an electrostatic interaction between His268 and Glu294 at the C_H_2 domain surface. However, this particular electrostatic interaction shows inter-species variation and, even in human IgG, does not always form. When the interaction is not formed, when the BC loop is disordered, or indeed in one structure, conformationally different, the conformation of the FG loop is not affected.

We have revealed the structure of the C_H_2 FG loop in native IgG4-Fc, unaffected by crystal packing, and instead propose that conformational differences between the IgG4 C_H_2 domain FG loop and that of IgG1 are due to the introduction of conformational flexibility through the presence of Gly327 and Ser331. Further structural data will be required to establish the essential residues that determine the FG loop conformation that we have observed.

### Conclusions

The first high-resolution structures for IgG4-Fc reported here have shed light on the unique functional features of this human antibody subclass. Variations in conformation at the C_H_3–C_H_3 interface involving Arg409 indicate how this residue, unique to IgG4, may destabilise interaction between the heavy chains and contribute to FAE. The FG loop in the C_H_2 domain of IgG4 is also seen to adopt a conformation distinct from that which is common to all other IgG subclasses and antibody classes (IgA, IgM, IgY); since this loop plays a key role in receptor and complement interactions, the non-inflammatory properties of IgG4 may now be understood. This conformation also precludes potentially stabilising interactions with the hinge region, which may further promote FAE. Finally, extensive intermolecular (crystal packing) contacts, including one interaction not previously observed in other IgG-Fc structures, may explain the tendency of IgG to aggregate. These results all have implications not only for the engineering of therapeutic monoclonal antibodies, but also for understanding the role of IgG4 antibodies in disease pathology.

## Materials and Methods

### Protein production

Serum-derived (sdFc) and recombinant (rFc) IgG4-Fc were prepared as described previously [39], [58]. Both proteins were dialysed into 20 mM Tris–HCl pH 8.

### Crystallisation

Crystals of both rFc and sdFc were grown at 291 K using a reservoir comprising 100 μL of 100 mM 4-morpholineethanesulfonic acid (MES) pH 6.5, 18–20% (w/v) polyethylene glycol 10,000, and protein concentrations of 3 mg/mL and 2.5 mg/mL, respectively. Drop sizes were 100 nL protein and 100 nL reservoir, and 300 nL protein and 300 nL reservoir, for the rFc and sdFc crystals, respectively. Crystals typically appeared after 2 days and were briefly cryoprotected in a solution of 100 mM MES pH 6.5, 20% (w/v) polyethylene glycol 10,000 and 20% (v/v) ethylene glycol before flash-cooling in liquid nitrogen.

### Data processing, structure determination and refinement

Data were collected at beamline I03 at the Diamond Light Source (Harwell, UK). Integration was performed with XDS as implemented in the *xia2* package [72], [73], and further processing was carried out using the CCP4 suite [74]. Both structures belong to space group *P*2_1_2_1_2_1_, with one molecule in the asymmetric unit, and were solved by molecular replacement using MOLREP [75]. The rFc structure (1.9 Å resolution) was solved using IgG4-Fc protein atoms from PDB ID 1ADQ [37] as a search model, and the sdFc structure (2.35 Å resolution) was solved used a partially refined recombinant IgG4-Fc structure. Refinement was performed with PHENIX [76], and manual model building was performed with Coot [77]. Carbohydrate residues were refined with 100% occupancy. 5% of reflections were assigned to the *R*_free_ reflection set using PHENIX, and TLS groups were assigned with PHENIX. Overall structure quality was assessed with MolProbity [78] and POLYGON [79] within PHENIX, and carbohydrate was assessed using CARP [80]. The final *R*_work_/*R*_free_ values were 17.01%/20.86% and 18.80%/23.94% for the rFc and sdFc structures, respectively. Full data processing and refinement statistics are provided in Table 1. For consistency, all buried surface area calculations were performed with CNS [81] using protein atoms only from entries available from the PDB. Figures were produced with PyMOL (The PyMOL Molecular Graphics System, Version 1.1r1, Schrödinger, LLC).

**Table 1:** Data processing and refinement statistics.

	Recombinant Fc (rFc)	Serum-derived Fc (sdFc)
*Data processing*		
Space group	*P*2_1_2_1_2_1_	*P*2_1_2_1_2_1_
Unit cell dimensions		
*a*, *b*, *c* (Å)	74.84, 78.97, 97.88	74.29, 80.74, 99.19
Resolution (Å)	74.84–1.90 (1.94–1.90)^a^	80.74–2.35 (2.45–2.35)^a^
No. of unique reflections^b^	46,409 (2957)^a^	25,471 (2828)^a^
Completeness (%)^b^	99.9 (99.9)^a^	99.7 (99.9)^a^
Multiplicity^b^	14.4 (14.4)^a^	10.7 (11.2)^a^
Mean [(*I*)/σ(*I*)]^b^	16.7 (2.1)^a^	14.4(1.6)^a^
*R*_merge_ (%)^b^	10.6 (154.5)^a^	10.0 (225.9)^a^
*R*_pim_ (%)^b^	2.9 (42.1)^a^	3.2 (69.6)^a^
Wilson *B*-factor (Å^2^)	26.9	56.9

*Refinement*		
*R*_work_/*R*_free_ (%)	17.01/20.86	18.79/23.81
RMSD		
Bond lengths (Å)	0.013	0.009
Bond angles (°)	1.490	1.123
Coordinate error (Å)	0.22	0.35
No. of atoms^c^		
Protein	3351	3195
Carbohydrate	209	209
Solvent	290	72
Other^d^	58	23
Average *B*-factor (Å^2^)		
Protein: C_H_2 A/B	32.96/46.16	55.2/76.4
Protein: C_H_3 A/B	33.28/33.04	61.84/67.8
Carbohydrate: A/B	45.83/59.60	53.2/83.1
Solvent	40.2	53.7
Other^d^	62.9	83.0
Ramachandran plot (%)^e^		
Favoured	99.53	97.82
Allowed	100	100

a Numbers in parentheses are for the highest resolution shell.

b Data scaled with Aimless [74].

c Includes alternative positions.

d Ethylene glycol and MES buffer.

e Ramachandran plot generated by MolProbity [78].

### Accession numbers

Coordinates and structure factors have been deposited at the PDB with IDs 4C54 and 4C554C544C55 for the rFc and sdFc structures, respectively.
