# Supplementary material for: Structural Determinants of Unique Properties of Human IgG4-Fc
Source: J Mol Biol. 2014 Feb 6;426(3):630–44. doi: 10.1016/j.jmb.2013.10.039 (PMC3905167; doi:10.1016/j.jmb.2013.10.039)
Supplement: Supplementary file 1 — Supplementary Figures S1 and S2. [file mmc1.pdf]

SUPPLEMENTARY INFORMATION

Fig. S1

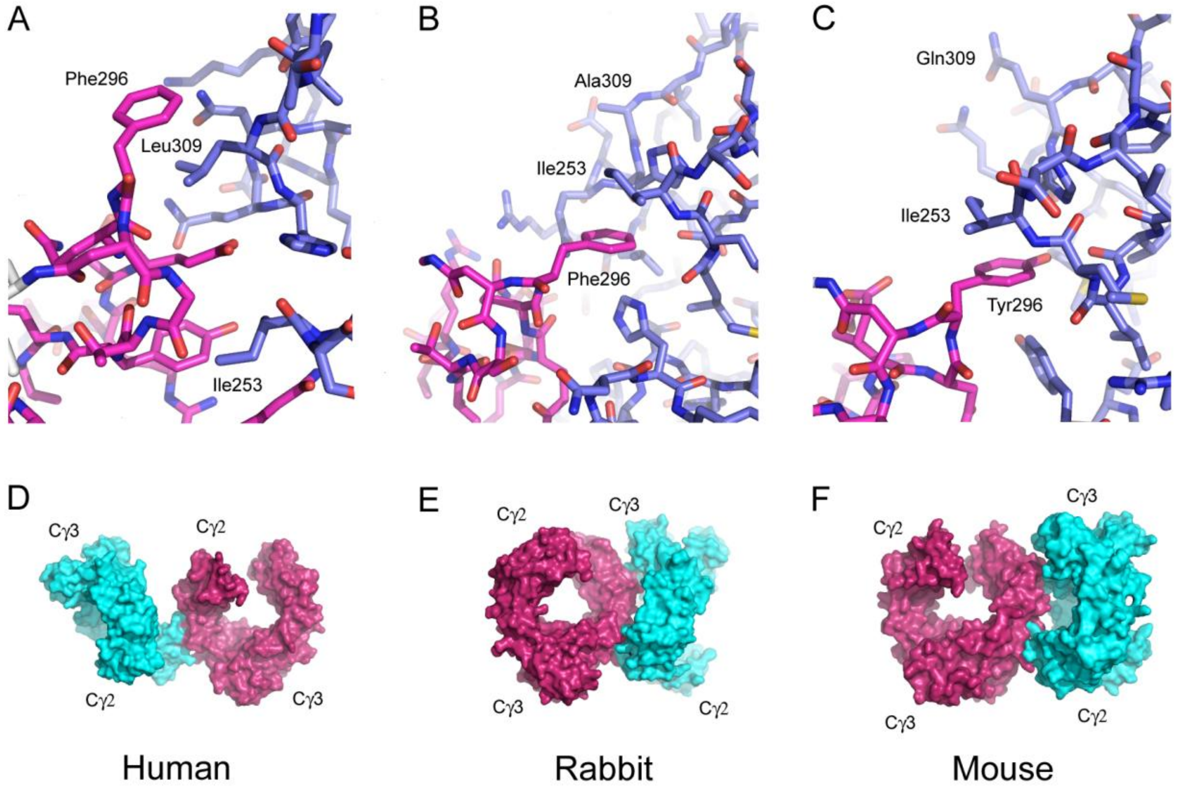

Fig. S1. Intermolecular interfaces involving residues 253 and 296 in human, rabbit and mouse IgG. (A) View of interface II identified in the human IgG4-Fc crystal structures. (*B*) View of an interface involving Ile253 and Phe296 in rabbit IgG (PDB ID **2VUO**). Unlike IgG4, residue 309 is an alanine and not involved in the interface. (*C*) View of an interface involving Ile253 and Tyr296 in mouse IgG2b (PDB ID **2RGS**). Unlike IgG4, residue 309 is a glutamine and not involved in the interface. (*D*) Orientation of two IgG4-Fc molecules interacting through interface II shown in (*A*). (*E*) Orientation of two rabbit IgG-Fc molecules interacting though the interface shown in (*B*). (*F*) Orientation of two mouse IgG2b-Fc molecules interacting through the interface shown in (*C*).

Fig. S2

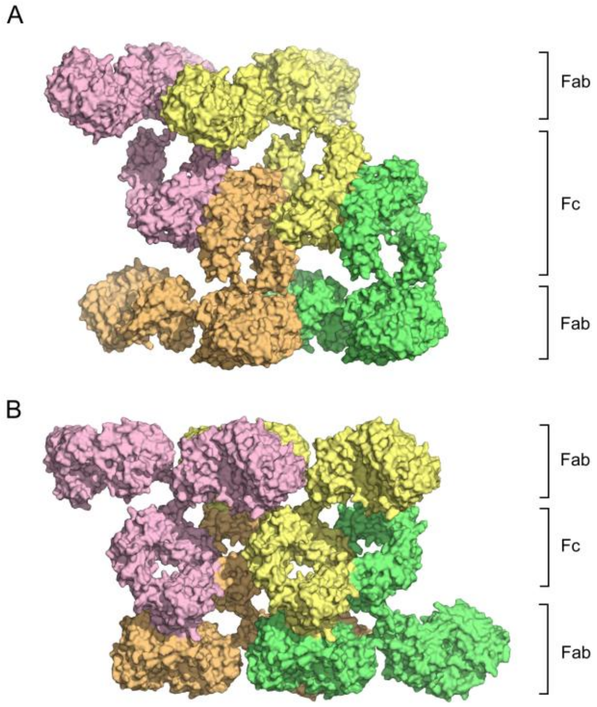

Fig. S2. Interfaces I and II can accommodate whole antibody structures. (*A*) An assembly utilising Interface I can be created using a whole antibody structure without any clashes between Fab and Fc components. (*B*) An assembly using Interface II can also be created with no clashes. The interaction is such that the top of the C_H2 domain (site containing Phe296) from chain A of one antibody molecule interacts with the side (site containing Ile253 and Leu309) of chain B from the second antibody molecule. In both figures, four whole antibody molecules are shown and the template structure used is that of murine IgG2a (PDB ID **1IGT**).
